# A Histopathological Comparison of Pulpotomy with Sodium Hypochlorite and Formocresol

**Published:** 2012-06-01

**Authors:** Roza Haghgoo, Farid Abbasi

**Affiliations:** 1. Department of Pediatric Dentistry, Dental School, Shahed University of Medical Sciences, Tehran, Iran; 2. Department of Oral Medicine, Dental School, Shahed University of Medical Sciences, Tehran, Iran

**Keywords:** Formocresol, Histological Techniques, Pulpotomy, Primary Teeth, Sodium Hypochlorite

## Abstract

**Introduction:**

Formocresol is widely used in primary teeth pulpotomies; however it is known to have several side effects. The purpose of this study was to assess pulpal changes of primary teeth pulps after pulpotomy with sodium hypochlorite and formocresol.

**Materials and Methods:**

In this randomized clinical trial, 22 teeth were selected. These teeth were randomly divided into 2 groups (formocresol; n=11 and sodium hypochlorite n=11). Two months post-operatively teeth were extracted and pulpal responses were evaluated by recording the degree of inflammation and extent of pulpal involvement. Dentinal bridge formation was also evaluated. Finally the data was analyzed with McNemartest.

**Results:**

The formocresol group demonstrated mild inflammation in 4 cases whereas sodium hypochlorite had mild inflammation in six cases. Severe inflammation was only found in 1 case in the sodium hypochlorite group, but it was present in 4 cases in the formocresol group. In sodium hypochlorite group there were no cases of necrosis, and dentinal bridge was found in 3 cases, unlike, the formocresol group which had necrosis but no dentinal bridge formation.

**Conclusion:**

Based on the results of this study sodium hypochlorite may be a suitable solution for conducting pulpotomy in primary teeth.

## Introduction

Pulpotomy is one of the most common ways to treat cariously exposed pulp and symptom-free primary teeth [1]. The rational is based on the healing ability of the radicular pulp tissue following amputation of the affected/infected pulp [2]. Pulpotomy is performed through 3 phases: devitalization of the coronal pulp, preservation and regeneration. The ideal pulp dressing material must be bactericidal, harmless to the pulp and surrounding structures, promote healing of the radicular pulp and not interfere with the physiological process of root resorption [3]. The most usual pulp dressing is formocresol (which devitalizes the pulp) which consists of 19% formaldehyde, 35% cresol, glycerin and water [4]. Success rate of formocresol pulpotomy is 70-98% [5][6][7][8]. However, several studies have reported its potential for the local/systemic side effects; i.e. local pulpal inflammation/necrosis, general cytotoxicity, mutagenic/carcinogenic effect, systemic disturbances, and immunologic responses [9]. Concerns have been expressed about effect of formocresol on the enamel structure of the permanent successors [10].

Sodium hypochlorite (SH) is a common agent for irrigation of root canal(s) and it is used for hemostasis, removal of debris and biofilm [11] as well as pulpotomy medicament; clinical and radiographic success rates of SH pulpotomy were reported to be 100% and 76%, respectively [12]. When considering the detrimental effects of formocresol and suitable properties of SH, it may be a wise to perform the pulpotomy of primary teeth with SH. Therefore, the purpose of this in vivo study was to evaluate pulp status after pulpotomy with SH and formocresol.

## Materials and Methods

This study was conducted at Shahed University Dental School, Tehran, Iran. The patients were recruited from children who were between 7-8 years and referred to pediatric department. Twenty-two canines (by split mouth design) that were due for extraction due to orthodontic reasons were selected. These teeth were sound or at least did not have any root resorption in their coronal two-thirds.

Written informed consent was obtained from the parents. The protocol was approved by the Ethics committee of Shahed University. The teeth were randomly allocated into 2 groups by a random number producing system: 11 teeth in formocresol group and 11 teeth in SH group. All pulpotomies were carried out by a pedodontist.

After administration of local anesthesia with lidocaine (Darou Pakhsh, Tehran, Iran) and rubber dam isolation, coronal access was created; the coronal pulp was removed with a spoon excavator. After pulp amputation, pulp chamber was rinsed with normal saline solution. Hemorrhage was controlled by placing a cotton pellet moistened in saline with slight pressure. In formocresol group, after hemostasis, small cotton pellet soaked in formocresol (SSA, Produits Dentaires, Switzer-land) was placed over the orifice of canal for 5 min; the orifice was then covered with ZOE (Produits Dentaires, Vevey, Switzerland) and filled with amalgam (Cavex Avalloy, Cavex Co., Holland).

In SH group, after hemostasis, small cotton pellet soaked in 5% SH (Pakshoma, Tehran, Iran) was placed over the canal orifice for 15 seconds; the orifice was then covered with ZOE and filled with amalgam.

After 2 months the teeth in the 2 groups were extracted. Serial sections were cut for H & E staining. A pathologist, who was not informed about study design, studied these sections. The evaluation was performed according to the criteria by Fuks et al. [13] as follows: 0=none/mild inflammation; 1=moderate inflammation; 2= severe inflammation; 3=necrosis; 4=abscess; and 5=resorption. In addition, the presence or absence of a dentin bridge was evaluated. Finally the data was analyzed by McNemar test.

## Results

**Figure 1:**
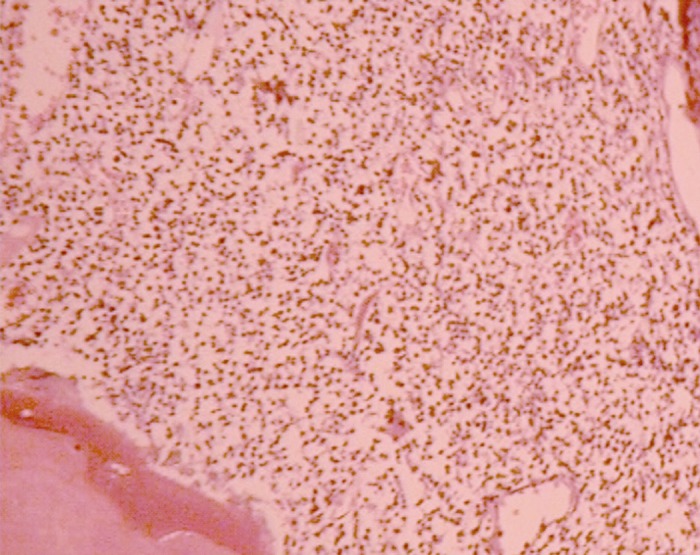
Severe inflammation 2 months after pulpotomy with formocresol (H and E×40)

**Figure 2:**
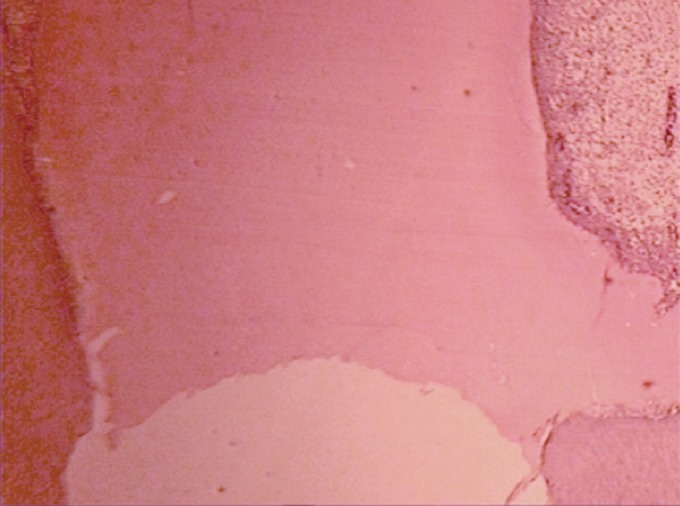
Dentinal bridge formation 2 months after pulpotomywith sodium hypochlorite (Hand E×40)

A total of eight children between the ages of 7-8 years participated in this in vivo study. The results of histopathologic evaluations as well as dentine bridge formation are shown in Table 1 (Figure 1 and Figure 2).

**Table 1:** Pulpal changes and dentinre bridge formation in the experimental groups

**Pulpal response**	**Sodium hypochlorite *(n)***	**Formocresol *(n)***	***P* value**
**Mild inflammation**	6	4	0.678
**Moderate inflammation**	4	3	0.900
**Severe inflammation**	1	4	0.375
**Necrosis**	0	5	-
**Abscess**	0	0	-
**Internal resorption**	2	0	-
**Dentin bridge**	3	0	-

## Discussion

Formocresol, a once popular pulpotomy agent in primary teeth, has several side effects [3]. SH is a hemostatic agent and can probably be used in pulpotomy of primary teeth [11]. The purpose of this histopathologic study was to evaluate the effect of SH as well as formocresol on the pulp. For the first time, the pulp status of primary teeth that underwent pulpotomy with SH and formocresol was compared; we had not found any previous studies that reported the histopathological status of the pulp, though there were studies looking at the success rates. Vargas et al. reported favorable success rate of pulpotomy with SH [12]. Our results showed that severe inflammation in formocresol is more than SH. Sodium hypochlorite can induce hemostasis and control the hemorrhage and therefore this may decrease potential for inflammation following pulpotomy. Four teeth in the formocresol group resulted in pulpal necrosis; however, no necrosis was seen in the SH group. Formocresol devitalizes the pulp and this phenomenon can induce necrosis.

Interestingly, in the SH group, three teeth had dentinal bridge formation. The preservation of pulp vitality in the SH group may explain how the pulps in this group can regenerate and form a dentinal bridge. Internal resorption was seen in 2 cases in SH group, but none in the formocresol group. The antimicrobial property of SH may also trigger internal resorption [14].

## Conclusions

Based on the results of this study SH may be suggested as a pulpotomy agent in primary teeth. As this study was only performed on 22 teeth, we suggest a more extensive study to be performed with greater histopathological samples. Moreover, the clinical and radiographic success rates of primary teeth pulpotomized with SH and formocresol should be evaluated.
